# Exosomes derived from MSCs exposed to hypoxic and inflammatory environments slow intervertebral disc degeneration by alleviating the senescence of nucleus pulposus cells through epigenetic modifications

**DOI:** 10.1016/j.bioactmat.2025.02.046

**Published:** 2025-03-20

**Authors:** Yongzhao Zhao, Longting Chen, Shuai Jiang, Zhenquan Wu, Qian Xiang, Jialiang Lin, Shuo Tian, Zhuoran Sun, Chuiguo Sun, Weishi Li

**Affiliations:** a Department of Orthopaedics, Peking University Third Hospital, Beijing, China; b Beijing Key Laboratory of Spinal Disease Research, Beijing, China; c Engineering Research Center of Bone and Joint Precision Medicine, Ministry of Education, Beijing, China

**Keywords:** Low back pain, Intervertebral disc degeneration, Cell senescence, Epigenetic modification, Exosomes, MicroRNAs

## Abstract

Intervertebral disc degeneration (IDD) is the leading cause of low back pain, which places heavy burdens on society and individuals. Surgical intervention is the conventional therapy for IDD, but patients who undergo surgery face relatively high risks of recurrence and complications. Therefore, a relatively less invasive and efficient treatment for IDD is urgently needed. In this study, we constructed a novel nanobiomaterial, named Hi-Exos, to slow IDD. Hi-Exos are exosomes derived from mesenchymal stem cells exposed to hypoxic and inflammatory environments. Hi-Exos could relieve the senescence of nucleus pulposus cells and slow IDD through an epigenetic modification mechanism by introducing the epigenetic factor miR-221-3p into senescent nucleus pulposus cells to reduce DDIT4 expression and inhibit the activation of NF-κB signalling pathway. This study provided a novel strategy for IDD treatment involving the use of Hi-Exos to deliver miR-221-3p to reduce the senescence of nucleus pulposus cells and repair IDD via epigenetic modifications.

## Highlights

• Hi-Exos was superior to Exos in relieving the senescence of nucleus pulposus cells and intervertebral disc degeneration.
• Epigenetic factor miR-221-3p played an important role in intervertebral disc degeneration.
• Hi-Exos transferred the miR-221-3p to inhibit the DDIT4/NF-κB pathway.
• Hi-Exos provided a novel strategy for intervertebral disc degeneration.

## Graphical abstract

**Figure:**
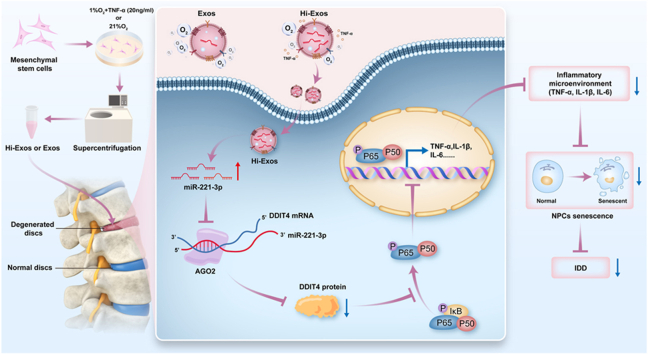

## 1 Introduction

Intervertebral disc degeneration (IDD) is the leading cause of low back pain, which places heavy burdens on society and individuals with related disabilities [1]. The intervertebral disc consists of the central gelatinous nucleus pulposus (NP), outer collagenous annulus fibrosus, and inferior and superior cartilage end plates [2]. Among the components of intervertebral discs, the central NP is the most important anatomical structure for maintaining the mechanical stability of the spine [2]. The conventional therapy for IDD includes the use of pain medications and resection of herniated discs [3]. However, the use of such medications only can relieve part pain symptoms but cannot prevent IDD progression, as a result, many patients eventually have to undergo surgeries. Surgical therapy for IDD can improve clinical symptoms through the removal of herniated intervertebral discs; however, patients face high risks of perioperative complications and recurrence [4]. Therefore, novel therapies for IDD urgently need to be raised to make up for the shortcomings of existing treatments for IDD.

Cell senescence is induced by several exogenous and endogenous stresses, such as inflammatory stimulation, oxidative stress, and DNA damage [5]. The main characteristics of cellular senescence are cell cycle arrest, activation of senescence-associated GLB1/β-galactosidase, and a senescence-associated secretory phenotype [5]. Cell senescence has been shown to play important roles in IDD, and inflammatory factors (e.g., TNF-α and IL-1β) are commonly used to induce the senescence of nucleus pulposus cells (NPCs) [6]. Although increasing attention has been given to the senescence of NPCs, we still know little about the underlying mechanisms relevant to this process.

Since mesenchymal stem cells (MSCs) possess multipotent differentiation potential, they hold promise in a variety of ageing-related diseases [7,8]. We previously reported that MSCs could alleviate the IDD by reducing the oxidative stress in NPCs [9]. However, the direct utilization of MSCs is partially limited by several associated risk factors, such as potential chromosomal variations and immune rejection [7,8]. Therefore, developing a superior treatment that possesses the therapeutic effect of MSCs while reducing the possible risks associated with the direct utilization of MSCs is vital. MSCs exert therapeutic effects mainly through paracrine mechanisms, including the secretion of exosomes [8]. Exosomes are 30–150 nm extracellular vesicles that can carry proteins, nucleic acids, and lipids, and they are secreted by parent cells [10]. Exosomes are cell-free, reducing immune rejection and tumor risks [10]. They are stable, easily stored, and efficiently deliver nucleic acids, mimicking stem cell effects without ethical concerns or storage limitations [11,12]. Many studies have demonstrated that MSC-derived exosomes can delay IDD [13]. Moreover, several strategies, such as treatment of MSCs with hypoxic conditions or inflammatory factors (e.g., TNF-α and IL-1β), have been used to increase the repair capacity of MSC-derived exosomes in human diseases because these strategies could regulate the cargos in exosomes [[14], [15], [16], [17], [18], [19]]. Hypoxic pretreatment could enhance the repair capacity of exosomes by increasing the amounts of some cargos in the exosomes, such as miR-205-5p [20], miR-214-3p [21], BNIP3 [14], and miR-21-5p [22]. The higher repair capacity of MSC exosomes pretreated with hypoxic pretreatment might be explained by that most MSCs are in a hypoxic ecological niche *in vivo,* and culturing MSCs in a low-oxygen environment is more closely aligned with their physiological environment [19]. Pretreatment with inflammatory factors (e.g. TNF-α, IL-1β, and LPS) is another important strategy to increase the repair capacity of exosomes by increasing the level of some cargos (e.g. LRP1 [16], miRNA-222-3p [18], and miR-21-5p [23]) to increase the immunomodulatory capacity [18]. However, to date, no study has explored the repair capacity of exosomes derived from MSCs exposed to combined hypoxic and inflammatory environments in the context of IDD treatment through alleviating the senescence of NPCs.

Epigenetic modification refers to the alteration of gene expression without altering the DNA sequence, which mainly includes changes in DNA methylation, histone modifications, and noncoding RNAs [24]. Epigenetic modification plays important roles in the pathogenesis of degenerative musculoskeletal disorders [25,26], for instance, we previously found that N6-methyladenosine-modified circCDK14 could promote the ossification of the ligamentum flavum via the epigenetic modulation [25]. MicroRNAs (miRNAs) are epigenetic regulators without protein-coding ability, and they can promote mRNA degradation or inhibit the translation of targeted genes by binding to their 3′-untranslated regions (UTRs) [27]. Many studies have shown that strategies involving pretreatment of MSCs benefit their repair capacity by changing the expression profile of miRNAs in their exosomes [19,28]. However, the alteration of miRNA profiles in exosomes has not been fully investigated in response to MSC exposure to hypoxic and inflammatory environments.

In this study, we discovered that exosomes derived from MSCs exposed to hypoxic and inflammatory environments (Hi-Exos) could significantly reduce the senescence of NPCs and improve outcomes for *in vivo* IDD experiments. Specifically, the exosomal miRNA profiles revealed that miR-221-3p level was significantly greater in the Hi-Exos than that in the Exos, which were derived from MSCs under normal culture conditions. Hi-Exos regulated the DDIT4/NF-κB signalling pathway to reduce the senescence of NPCs via transferring epigenetic factor miR-221-3p to senescent NPCs. Our findings indicated that Hi-Exos constituted a novel and promising therapy for IDD.

## 2 Materials and methods

### 2.1 Human NP tissue samples

The degenerated NP tissues were obtained from patients with symptomatic IDD, and the normal NP tissues were obtained from patients with scoliosis without IDD. The Ethical approval was obtained from the Ethics Committee of Peking University Third Hospital, and informed consent was obtained from all patients involved in this study.

### 2.2 Culture of rat NPCs and bone MSCs

Twelve-week-old Sprague–Dawley rats were purchased from the Laboratory Animal Center of Peking University Health Science Center (Beijing, China). Briefly, the rat NP tissues were isolated, cut into pieces, and digested in 0.2 % type II collagenase (Solarbio, USA) for 6 h at 37 °C with 5 % CO_2_. The digested tissues were subsequently washed with phosphate-buffered saline (PBS) three times, centrifuged at 1000 rpm for 5 min, and subsequently cultured in DMEM/F12 supplemented with 10 % foetal bovine serum (FBS) (Thermo Fisher Scientific, USA) and 1 % penicillin/streptomycin at 37 °C with 5 % CO_2_. Primary rat bone MSCs were purchased from a commercial company (Procell, China) and cultured in α-MEM supplemented with 10 % FBS and 1 % penicillin–streptomycin at 37 °C with 5 % CO_2_. For NPCs and MSCs, the culture medium was changed every three days, and the cells were passaged at a 1:3 ratio when they reached 70–80 % confluence. Cells from passages 3–5 were used for further experiments. NPC senescence was induced by exposing the cells to 20 ng/ml TNF-α (PeproTech, USA) for 72 h.

Four types of exosomes were derived from MSCs pretreated with hypoxia (1 % O_2_) or inflammatory factor TNF-α (20 ng/ml) (PeproTech, USA) for 12 h, and the details were listed as follows: (1) Exos were derived from MSCs cultured with 21 % O_2_ (normoxia); (2) H-Exos were derived from MSCs cultured with 1 % O_2_ (hypoxia); (3) I-Exos were derived from MSCs cultured with 21 % O_2_ (normoxia) and TNF-α; (4) Hi-Exos were derived from MSCs cultured with 1 % O_2_ (hypoxia) and TNF-α. For exosome interventions in cell experiments, the exosomes were added to culture dishes to maintain the exosome concentration of 100 μg/ml [14,29].

### 2.3 RNA interference, miRNA mimics or inhibitors, and plasmid transfection

The siRNAs for DDIT4 or AGO2, mimics for miR-221-3p, and inhibitors for miR-221-3p were synthesized by Sangon Biotech (Shanghai, China), and detailed sequences are listed in Supplementary Table 1. The plasmids used for overexpressing DDIT4 were constructed by GenePharma (Shanghai, China). The siRNAs targeting DDIT4 or AGO2, miR-221-3p mimics, miR-221-3p inhibitors, and DDIT4 overexpression vectors were transfected into rat NPCs or MSCs with Lipofectamine 3000 (Invitrogen, USA) according to the manufacturer's protocol. The transfection efficiency was confirmed by qRT-PCR assays or Western blots 48 h after transfection.

### 2.4 Extraction, identification, and uptake of exosomes

When the MSCs reached 70–80 % confluence, α-MEM supplemented with 10 % exosome-free FBS and 1 % penicillin/streptomycin was added for 12 h of culture under specific conditions. The conditioned culture medium was then harvested and filtered through a 0.22 μM strainer (Thermo Fisher Scientific, USA). The cell mixture was subsequently centrifuged at *300 × g* for 10 min, *2000 × g* for 20 min, 10,*000 × g* for 30 min, and 110,*000 × g* for 70 min to obtain exosome pellets. The obtained purified exosomes were resuspended in PBS. For the identification of exosomes, their morphology was observed via transmission electron microscopy, the particle size distribution was evaluated via nanoparticle tracking analysis, and exosome markers (TSG101, HSP70, and calnexin) were evaluated via Western blot. For the uptake of exosomes, NPCs were seeded in six-well plates at a density of 1 × 10^5^ cells per well and cultured for 24 h. Exosomes were labeled with PKH67 (Solarbio, China) according to the manufacturer's protocol and incubated with NPCs for 6 h. Then, the cells were fixed and stained with DAPI solution (Solarbio, China) and observed under a fluorescence microscope (Nikon, Japan).

### 2.5 RNA isolation and quantitative real-time PCR (qRT‒PCR)

Total RNA was extracted from MSCs, NPCs, and exosomes with Trizol methods (Thermo Fisher Scientific, USA). Reverse transcription was performed on extracted RNA via commercial kits (Accurate Biology, China). RT‒PCR assays were conducted using the SYBR Green Premix Kit (Accurate Biology, China) on the QuantStudio 5.0 version (Thermo Fisher Scientific, USA). The PCR program was as follows: initial denaturation at 95 °C for 30 s, 40 cycles of denaturation at 95 °C for 5 s, annealing at 60 °C for 30 s, extension at 72 °C for 60 s, and a final extension at 72 °C for 10 min. The relative transcription levels were standardized to those of GAPDH for mRNA and U6 for miRNA via the 2^−ΔΔCt^ method. The details of the primers used for the qRT‒PCR assays are listed in Supplementary Table 1.

### 2.6 Western blot analysis

Cells, exosomes, and tissues were lysed with RIPA buffer (Solarbio, China). The lysis mixture was them heated at 95 °C for 20 min after loading buffer (Solarbio, China) was added. Then, the lysis mixture was separated on a 12 % gel (EpiZyme, China) and transferred to polyvinylidene fluoride membranes (Millipore, USA) via a Bio-Rad device (Bio-Rad, USA). The membranes were blocked with 5 % skim milk powder (Epizyme, China) for 2 h and incubated with primary antibodies for 2 h at room temperature. The polyvinylidene fluoride membranes were incubated with HRP-conjugated secondary antibodies (Solarbio, China) for 1 h at room temperature. Finally, the protein bands were visualized with an iBright CL1000 device (Thermo Fisher Scientific, USA). The primary antibodies used were as follows: anti-p21 (1:1000, ab109199, Abcam, USA), anti-DDIT4 (1:2000, 10638-1-AP, Proteintech, USA), anti-p65 (1:1000, 8242T, CST, USA), anti-p-p65 (1:1000, 3033, CST, USA), acti-AGO2 (1:1000, ab186733, Abcam, USA), and anti-Actin (1:2000, ab8227, Abcam, USA). The quantitative analysis of the Western blot data was conducted via ImageJ software.

### 2.7 Dual-luciferase reporter assay

293T cells were seeded at 1×10^5 cells/well in a 24-well plate the night before transfection to achieve 70-80% confluence. A mixture of 2 μL miR-221-3p mimics or NC mimics (100 nM final concentration) and 1 μg wild type (WT) or mutant type (MUT) plasmids (DDIT4-WT-psiCHECK2 or DDIT4-MUT-psiCHECK2) (GeneSeed, China) was prepared in 50 μL serum-free medium. Separately, 2 μL lipofectamine 3000 (Invitrogen, USA) was diluted in 50 μL serum-free medium, incubated for 5 min at room temperature, and then combined with the miRNA/plasmid mixture. The mixture was incubated for 15 min at room temperature to allow complex formation. A total of 100 μL of the complexes was added to each well containing 300 μL complete medium, gently mixed, and incubated at 37°C, 5% CO2 for 5-6 h. The medium was replaced with fresh complete medium, and culturing was continued. Forty-eight hours later, luciferase activity was examined using a Dual Luciferase Reporter Assay Kit (Beyotime, China) according to the manufacturer's instructions.

### 2.8 EdU staining assay

An EdU staining assay was used to examine the proliferation status of NPCs. NPCs were seeded in a 12-well plate at a density of 2 × 10^5^ cells per well. A solution of 25 μmol/L EdU from a commercial kit (Beyotime, China) was added to the culture medium. The plate was subsequently incubated in a cell culture incubator for 2 h, and the subsequent steps were conducted according to the manufacturer's protocol. Finally, the results were observed under a fluorescence microscope (Nikon, Japan). The EdU-positive cells were analysed via ImageJ software.

### 2.9 Senescence-associated beta-galactosidase (SA-β-gal) staining assay

SA-β-gal staining assays were conducted using a commercial kit (Solarbio, China) according to the manufacturer's instructions. Briefly, the NPCs were washed three times with PBS. Then, 0.5 ml of prepared staining working solution was added to each well, and the plates were incubated for 12 h at 37 °C in a CO_2_-free incubator. The images were captured via a fluorescence microscope (Nikon, Japan) and statistically analysed by calculating the positive area ratio via the ImageJ software.

### 2.10 RNA pull-down and RNA immunoprecipitation (RIP) assay

RIP assays were conducted via a specific RNA-binding protein immunoprecipitation kit (GeneSeed, China) according to the manufacturer's instructions. The coprecipitated protein AGO2 was identified via the Western blots, and the coprecipitated RNA was evaluated via the qRT-qPCR.

### 2.11 Immunofluorescence assay

Slides containing NPCs were initially treated with 4 % paraformaldehyde at room temperature for 30 min. Then, the cells were permeabilized with 0.3 % Triton X-100 (Solarbio, China) for 15 min and blocked with immunostaining blocking solution (Beyotime, China) for 1 h. The cells were incubated with an anti-p65 antibody (1:1000, 8242T, CST, USA) overnight at 4 °C. The next day, the slides of NPCs were incubated with the corresponding Alexa Fluor 488-labeled Goat Anti-Rabbit IgG (H + L) (1:500, Beyotime, China) in the dark at room temperature for 1 h. Then, the slides were stained with DAPI (Beyotime, China) in the dark at room temperature for 10 min. Finally, the slides were washed three times with PBS for 10 min each time. The cells on the slides were then observed under a fluorescence microscope (Nikon, Japan).

### 2.12 Histological and immunohistological analysis

The samples were harvested and fixed with 4 % paraformaldehyde, decalcified with 10 % ethylenediaminetetraacetic acid solution, and embedded in paraffin. The samples were cut into 5-μm sections, and the histological features were examined by haematoxylin and eosin (H&E), safranin-O-fast green (SO&FG), and alcian blue staining. The tissue sections were incubated with the primary antibodies anti-MMP13 (1:300, A11148, ABclonal, China) at 4 °C overnight. Then, the sections were washed with PBS and incubated with a secondary antibody for 60 min at room temperature (1:200, PR30011, Proteintech, USA). Histologic images were evaluated following the histologic grading scale criteria [30], and the relative MMP13 protein expression was assessed using the ImageJ software.

### 2.13 *In vivo* rat IDD model and intradiscal injection

Protocols were approved by the Institutional Animal Care and Use Committee at Peking University Health Science Center. Twelve-week-old, 200 g ± 20 g male Sprague Dawley rats were obtained from the Laboratory Animal Center, Peking University Health Science Center. The rats were raised in a standard specific pathogen-free environment under a 12-h light/12-h dark cycle at 21 °C. The animals were randomly and blindly assigned to groups for *in vivo* experiments. The *in vivo* IDD model was established as previously reported [6,31]. After the anaesthetization with tribromoethyl alcohol (10 ml/kg) via intraperitoneal injection, the coccygeal intervertebral disc needle puncture IDD model was established with a 22G needle at the Co6‒7 or Co7‒8 segment. Seventy-two *in vivo* rats were randomly divided into six groups (Sham, IDD treated with PBS, IDD treated with Exos injection, IDD treated with Hi-Exos injection, IDD treated with Hi-Exos^NC inhibitors^ injection, and IDD treated with Hi-Exos^miR−221−3p inhibitors^ injection). Exosomes were injected into operated discs at a volume of 5 μl (1 μg/μl) once a week for four weeks [14].

### 2.14 RNA sequencing and bioinformatic analysis

For exosomal miRNA sequencing, exosomes were obtained from serum-free cell supernatants via the ultracentrifugation method. Total RNA was isolated and purified with the Exosomal RNA Isolation Kit (NGB-58000, CA) according to the manufacturer's protocol and then was delivered to LC-Bio to conduct miRNA sequencing, which was performed via single-end 50 bp for Illumina HiSeq 2500 according to the vendor's recommended protocol. The differential expression of miRNAs on the basis of normalized deep-sequencing counts was determined via Student's *t*-test. To predict the genes targeted by the differentially expressed miRNAs, TargetScan 5.0 and miRanda 3.3a were used to identify miRNA binding sites. The GO terms (http://www.geneontology.org/) and KEGG pathway terms (http://www.genome.jp/kegg/) of the miRNA targets were annotated.

For RNA sequencing, total RNA was isolated and purified using TRIzol reagent (Invitrogen, USA) according to the manufacturer's procedure. The RNA lysis mixtures were used to construct the library, and 2 × 150 bp paired-end sequencing (PE150) was performed on an Illumina NovaSeq™ 6000 (LC-Bio, China) according to the vendor's recommended protocol. The differentially expressed mRNAs with a fold change >1.8 or <0.56 and according to a parametric F test comparing nested linear models (P < 0.05) were selected via the R package edgeR. The KEGG enrichment (http://www.genome.jp/kegg/) of the differentially expressed mRNAs were annotated. The gene set enrichment analysis (GSEA) of differentially expressed mRNAs was conducted using the GSEA 4.2.3 software (Massachusetts Institute of Technology, USA).

### 2.15 Statistical analysis

All the data are presented as the means ± standard deviations. Two-tailed unpaired Student's *t*-test, or Welch's *t*-test, or one-way ANOVA followed by the Tukey–Kramer test was used to evaluate the statistical significance of any differences. P values of less than 0.05 were considered statistically significant. All experiments were independently repeated at least three times. All analyses were performed via GraphPad Prism 9.0 (GraphPad Software, USA).

## 3 Results

### 3.1 Senescence of NPCs in IDD

**Fig. 1:**
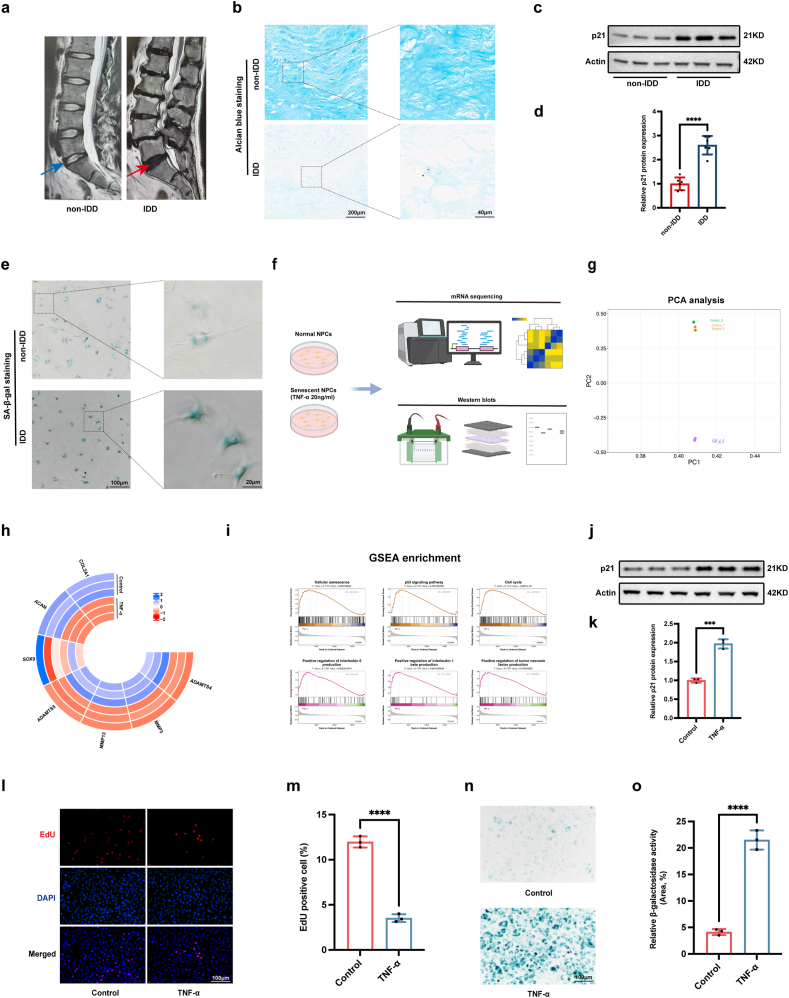
Senescence of NPCs is the main feature of IDD. a, Imaging characteristics of intervertebral discs on non-IDD or IDD patients on MRI; b, Alcian blue staining of NP tissues in non-IDD or IDD tissues; c, d, Protein expression of p21 in non-IDD or IDD tissues detected by Western blots; e, SA-β-gal staining of NPCs derived from non-IDD or IDD tissues; f, mRNA sequencing and Western blots to detect the mRNA and protein change between normal NPCs and senescent NPCs; g, PCA of differently expressed genes between normal NPCs and senescent NPCs; h, mRNA levels of extracellular matrix synthesis-related markers between normal and senescent NPCs, including COL2A1, ACAN, SOX9, ADAMTS5, MMP13, MMP3, and ADAMTS4; i, GSEA enrichment of differently expressed genes between normal and senescent NPCs; j,k, Protein levels of p21 between normal and senescent NPCs; l,m, EdU assay to detect the cell proliferation of normal and senescent NPCs; n,o, SA-β-gal staining of normal and senescent NPCs. ∗∗∗P < 0.001 and ∗∗∗∗P < 0.0001.

The IDD is the most common degeneration of the spine (Fig. 1a). Alcian blue staining presented the decreased blue staining for proteoglycan and collagen contents in degenerative NP tissues compared to normal NP tissues (Fig. 1b). Senescence of NPCs is an important feature of IDD [6,32,33]. To further analyse the senescence of NPCs during IDD, we detected the expression level of the senescence-related marker p21 in degenerative or normal NP tissues, and discovered that the p21 protein level was significantly greater in degenerated NP tissues than in normal NP tissues (Fig. 1c and d). Moreover, the SA-β-gal staining assay showed that SA-β-gal activity was obviously greater in degenerative NP tissues than in normal NP tissues (Fig. 1e). Inflammation is a vital contributor to IDD, therefore, we used TNF-α (20 ng/ml) to construct an *in vitro* model of NPC senescence [6]. To determine the change of gene expression in senescent NPCs, we conducted the transcriptome mRNA sequencing and Western blot (Fig. 1f), and the principle component analysis showed that NPCs treated with TNF-α were significantly clustered apart from the corresponding normal NPCs (Fig. 1g). In NPCs treated with TNF-α, genes related to extracellular matrix synthesis (ACAN, COL2A1, and SOX9) were decreased, but genes related to extracellular matrix catabolism (MMP3, MMP13, ADSTM4, and ADSTM5) were increased (Fig. 1h). The GSEA showed that treatment of the NPCs with TNF-α was accompanied with several senescence-related pathway: Cellular senescence, p53 signaling pathway, Cell cycle, Positive regulation of IL-6 production, Positive regulation of IL-1β production, and Positive regulation of TNF-α production (Fig. 1i). Moreover, Western blot assay results revealed that p21 was significantly elevated in TNF-α-treated senescent NPCs compared with control NPCs (Fig. 1j and k). The results of the EdU staining assay indicated there was less proliferation of TNF-α-treated NPCs than control NPCs (Fig. 1l and m). In addition, the SA-β-gal staining assay revealed that SA-β-gal activity was significantly greater in TNF-α-treated NPCs than in control NPCs (Fig. 1n and o). These data suggest that NPC senescence is an important feature of IDD and that TNF-α treatment can successfully simulate the senescence of NPCs in the context of human IDD.

### 3.2 Characterization of Exos, H-Exos, I-Exos, and Hi-Exos

**Fig. 2:**
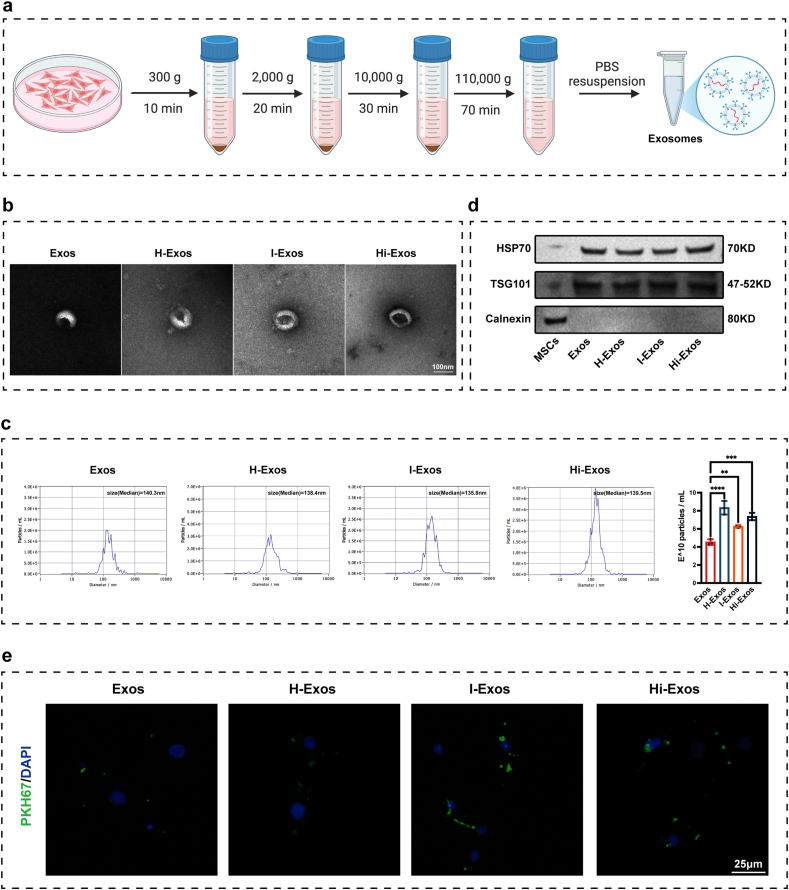
Extraction, identification, and uptake of exosomes derived from MSCs. a, Extraction of exosomes using the ultracentrifugation method; b, Transmission electron microscopy images of exosomes; c, NTA analysis for particle size distribution of exosomes; d, Western blots for exosome markers HSP70, TSG101, and Calnexin. e, Uptake of PKH67-labeled exosomes by NPCs. ∗∗P < 0.01, ∗∗∗P < 0.001, and ∗∗∗∗P < 0.0001.

Exos, H-Exos, I-Exos, and Hi-Exos were extracted using the ultracentrifugation method (Fig. 2a). All of Exos, H-Exos, I-Exos, and Hi-Exos had saucer-like shapes (Fig. 2b), and most had diameters that were between 30 and 150 nm, which indicated that there was no significant difference in morphology or diameter among four types of exosomes (Fig. 2c). But the secretion volume of exosomes was significantly different among four types of exosomes, and the secretion volume of Hi-Exos was significantly higher than Exos (Fig. 2c). Positive markers (TSG101, and HSP70) for exosomes were detected for all exosomes; in contrast, the negative marker Calnexin was not detected in any exosome (Fig. 2d). To confirm the uptake of exosomes by NPCs, PKH67-labeled exosomes were co-incubated with NPCs. Immunofluorescence analysis demonstrated that all four types of exosomes were successfully internalized by NPCs (Fig. 2e).

### 3.3 Hi-Exos reduce the senescence of NPCs through the transfer of miRNAs

**Fig. 3:**
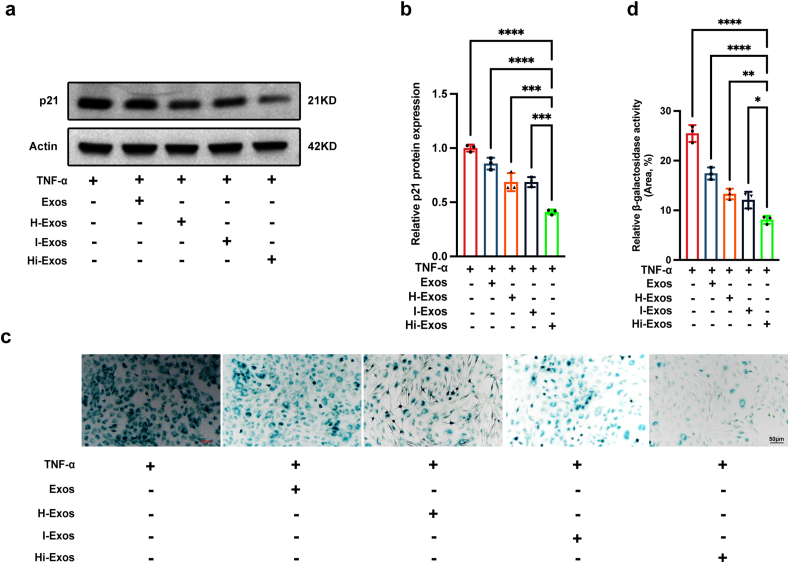
Efficacy of Hi-Exos in relieving the senescence of NPCs. a-b, Western blots to detect the protein level of p21 after the intervention of Exos, H-Exos, I-Exos, or Hi-Exos; c-d, SA-β-gal staining to detect the SA-β-gal activity after the intervention of Exos, H-Exos, I-Exos, or Hi-Exos. ∗P < 0.05, ∗∗P < 0.01, ∗∗∗P < 0.001, and ∗∗∗∗P < 0.0001.

The impact of four types of exosomes on the senescence of NPCs was assessed via Western blot analysis for p21 and SA-β-gal staining assays. The results showed that all exosomes could significantly reduce the p21 protein levels (Fig. 3a and b) and decreased the SA-β-gal activity of senescent NPCs (Fig. 3c and d). Moreover, compared with Exos, H-Exos, and I-Exos, Hi-Exos more strongly reduced p21 protein levels (Fig. 3a and b) and decreased SA-β-gal activity (Fig. 3c and d). Therefore, *in vitro* experiments revealed that Hi-Exos had a greater capacity than Exos to reduce the senescence of NPCs.

**Fig. 4:**
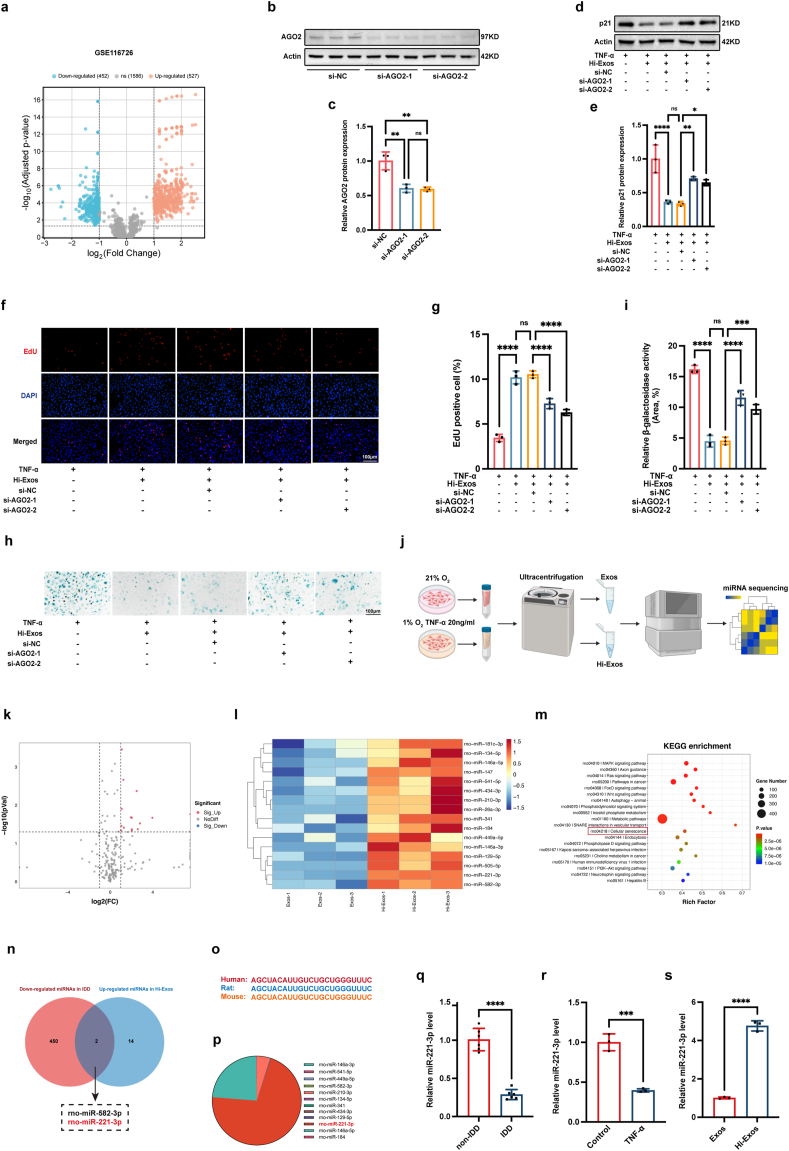
Hi-Exos relieved the senescence of NPCs through transferring the miRNAs. a, Volcano plot of differently expressed miRNAs between normal and degenerative NP tissues; b,c, Protein level of AGO2 after the transfection of siRNAs; d-i, Western blots to detect the protein level of p21 (d,e), EdU assay to detect the cell proliferation (f,g), and SA-β-gal staining to detect the SA-β-gal activity (h,i) after the intervention of Hi-Exos and knockdown of AGO2; j, Schematic diagram of miRNA sequencing in Exos and Hi-Exos. k-m, Volcano plot (k), heat map (l), and function enrichment (m) of differently expressed miRNAs between Exos and Hi-Exos; n, Intersection between downregulated miRNAs in degenerated NP tissues and upregulated miRNAs in Hi-Exos; o, Conservation of miR-221-3p among different species; p, Relative abundance of differently expressed miRNAs in Hi-Exos; q-s, miR-221-3p level in non-IDD or IDD tissues (q), normal NPCs or senescent NPCs treated with TNF-α (r), and Exos or Hi-Exos (s). ns, not significant (P < 0.05); ∗P < 0.05, ∗∗P < 0.01, ∗∗∗P < 0.001, and ∗∗∗∗P < 0.0001.

As important epigenetic factors, miRNAs play important roles in IDD. We reanalysed the GSE116726 data, which revealed the miRNA profiles of degenerative and normal NP tissues [34] to determine the miRNA differences between the two profiles. We discovered that 452 miRNAs were downregulated (fold change <0.50, adjusted P < 0.05) in IDD, which indicated that some miRNAs might play important roles in preventing IDD (Fig. 4a). Previous studies have demonstrated that MSC-derived exosomes perform repair functions predominantly through carrying cargos, such as miRNAs, mRNAs, and proteins [13]. Considering the important roles of miRNAs in IDD, we speculated that Hi-Exos might reduce the senescence of NPCs via the introduction of miRNAs. miRNAs are classical and important epigenetic regulators that promote mRNA degradation or inhibit protein translation by targeting complementary sequences located in the 3′-UTR of mRNAs; moreover, they perform these functions with the assistance of the RNA-induced silencing complex (RISC) [[35], [36], [37]]. Because AGO2 is the key component of RISCs [35], we used siRNAs to reduce the AGO2 level in senescent NPCs supplemented with Hi-Exos. The efficacy of siRNAs targeting AGO2 was verified by Western blot analysis (Fig. 4b and c). Hi-Exos obviously reduced the expression of p21 (Fig. 4d and e), promoted cell proliferation (Fig. 4f and g), and decreased the SA-β-gal activity (Fig. 4h and i) of senescent NPCs; however, these protective effects were compromised by the knockdown of AGO2. Taken together, our findings indicated that Hi-Exos might reduce the senescence of NPCs mainly through the transfer of miRNAs. To further determine the miRNAs in the exosomes, we extracted the Exos and Hi-Exos to conduct the miRNA sequencing (Fig. 4j). The heatmap (Fig. 4k) and volcano (Fig. 4l) plots revealed that a total of 16 miRNAs were significantly increased in Hi-Exos compared to Exos. To explore the biological functions of miRNAs in exosomes, we conducted the function enrichment analyses of the miRNA-targeted genes. The results revealed that differentially expressed miRNAs between Hi-Exos and Exos were associated with the cell senescence (Fig. 4m).

### 3.4 Hi-Exos reduced the senescence of NPCs through the transfer of miR-221-3p

To determine the potential miRNAs related to the repair capacity of Hi-Exos in IDD, we compared the upregulated miRNAs in Hi-Exos (fold change >2, P < 0.05) and downregulated miRNAs in degenerative NP tissues (fold change <0.50, P < 0.05), as a result, 2 miRNAs of interest (miR-582-3p and miR-221-3p) were obtained (Fig. 4n). We paid special attention to miR-221-3p because of its high degree of conservation among different species (Fig. 4o) and relatively high abundance (Fig. 4p). Further experiments confirmed that the level of miR-221-3p was significantly lower in degenerative NP tissues (Fig. 4q) and TNF-α-treated NPCs (Fig. 4r) than in normal NP tissues and control NPCs, respectively. But the expression level of miR-221-3p was obviously higher in Hi-Exos than in Exos (Fig. 4s).

**Fig. 5:**
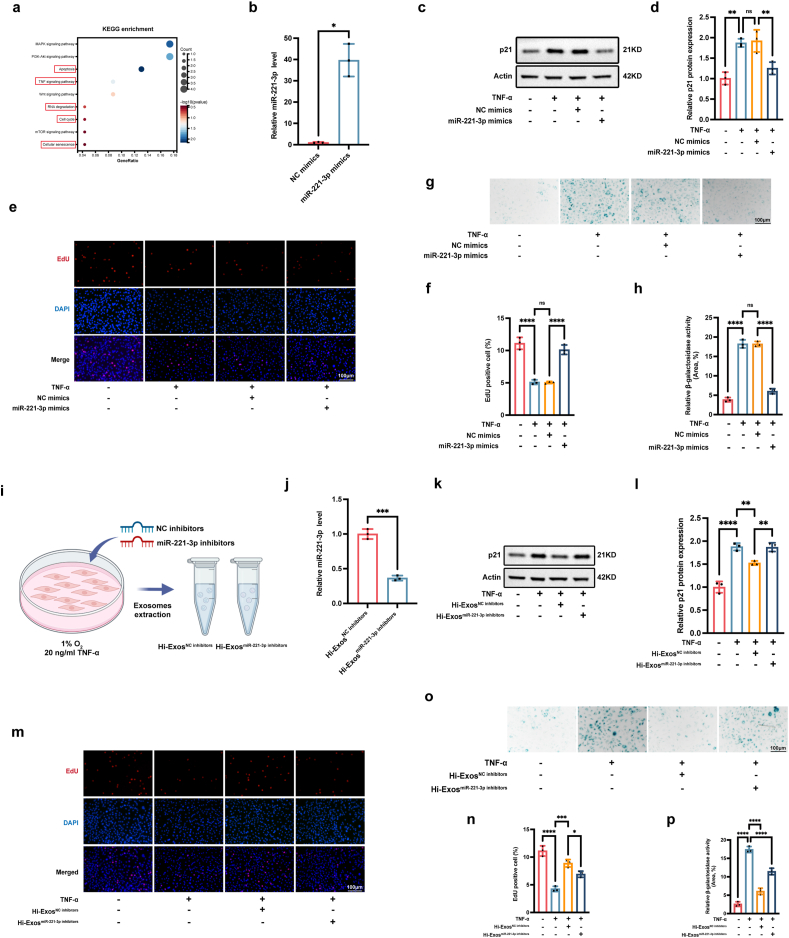
Hi-Exos relieved the senescence of NPCs through transferring the miR-221-3p. a, Function enrichment of miR-221-3p targeted genes; b, miR-221-3p level after the transfection of miR-221-3p mimics; c-h, Western blots to detect the protein level of p21 (c,d), EdU assay to detect the cell proliferation (e,f), and SA-β-gal staining to detect the SA-β-gal activity (g,h) after the transfection of miR-221-3p mimics; i, Schematic diagram of the acquisition of Hi-Exos^NC inhibitors^ or Hi-Exos^miR−221−3p inhibitors^; J, miR-221-3p level in Hi-Exos^NC inhibitors^ or Hi-Exos^miR−221 inhibitors^; k-p, Western blots to detect the protein level of p21 (k,l), EdU assay to detect the cell proliferation (m,n), and SA-β-gal staining to detect the SA-β-gal activity (o,p) after the intervention of Hi-Exos^NC inhibitors^ or Hi-Exos^miR−221−3p inhibitors^. ns, not significant (P < 0.05); ∗P < 0.05, ∗∗P < 0.01, ∗∗∗P < 0.001, and ∗∗∗∗P < 0.0001.

To further explore the biological roles of miR-221-3p in IDD, the function enrichment analysis of miR-221-3p targeted genes was conducted, and the following functions were enriched: apoptosis, TNF-α signalling pathway, RNA degradation, cell cycle, and cellular senescence (Fig. 5a). To further determine the biological roles of miR-221-3p in senescent NPCs, miR-221-3p mimics were transfected into NPCs, and the transfection efficacy was assessed via a qRT-PCR assay (Fig. 5b). Western blot analysis revealed that miR-221-3p overexpression obviously decreased p21 protein level (Fig. 5c and d), promoted cell proliferation (Fig. 5e and f), and reduced SA-β-gal activity (Fig. 5g and h) in senescent NPCs treated with TNF-α. Therefore, miR-221-3p clearly reduced the senescence of NPCs induced by TNF-α. To confirm that the repair capacity of Hi-Exos relying on the transfer of miR-221-3p to senescent NPCs, we introduced negative control or miR-221-3p inhibitors to MSCs exposed to hypoxic and inflammatory environments, enabling us to obtain Hi-Exos^NC inhibitors^ and Hi-Exos^miR−221−3p inhibitors^ (Fig. 5i). The qRT‒PCR results revealed that the miR-221-3p level in the Hi-Exos^miR−221−3p inhibitors^ group was obviously lower than that in the Hi-Exos^NC inhibitors^ Group (Fig. 5j). Then, we used Hi-Exos^NC inhibitors^ and Hi-Exos^miR−221−3p inhibitors^ to treat senescent NPCs. Compared with Hi-Exos^miR−221−3p inhibitors^, Hi-Exos^NC inhibitors^ had better repair capacity in terms of reducing p21 protein levels (Fig. 5k and l), promoting cell proliferation (Fig. 5m and n), and decreasing SA-β-gal activity (Fig. 5o and p). In summary, our findings demonstrated that Hi-Exos alleviate NPC senescence via the delivery of miR-221-3p.

### 3.5 DDIT4 was the downstream target of miR-221-3p

**Fig. 6:**
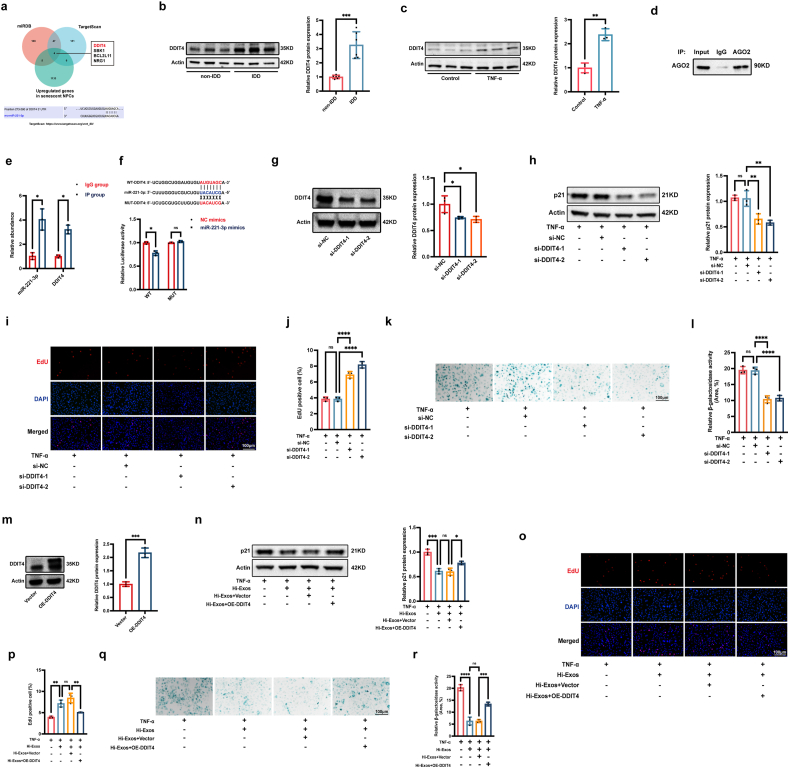
Hi-Exos reduced the senescence of NPCs by transferring the miR-221-3p to decrease DDIT4 expression. a, Bioinformatic analysis to determine the targeted genes of miR-221-3p; b, Protein level of DDIT4 in non-IDD tissues and IDD tissues; c, Protein level of DDIT4 in normal NPCs and senescent NPCs treated with TNF-α; d,e, Western blots to detect the AGO2 (d) and qRT-PCRs to detect the miR-221-3p and DDIT4 expression level in RIP analysis (e); f, Dual-luciferase reporter assay to determine the binding sites between miR-221-3p and DDIT4; g, Protein level of DDIT4 after the transfection of siRNAs; h-l, Western blots to detect the protein level of p21 (h), EdU assay to detect the cell proliferation (i,j), and SA-β-gal staining to detect the SA-β-gal activity (k,l) after the knockdown of DDIT4 in senescent NPCs induced by TNF-α; m, Western blots to detect the protein level of DDIT4 after the overexpression of DDIT4; n-s, Western blots to detect the protein level of p21 (n), EdU assay to detect the cell proliferation (o,p), and SA-β-gal staining to detect the SA-β-gal activity (q,r) in rescue experiments between Hi-Exos and DDIT4 overexpression. ns, not significant (P < 0.05); ∗P < 0.05, ∗∗P < 0.01, ∗∗∗P < 0.001, and ∗∗∗∗P < 0.0001.

The targets of miR-221-3p were predicted via the TargetScan database (https://www.targetscan.org/vert_80/) and the miRDB database (https://mirdb.org/). We compared the predicted targets with upregulated genes (fold change >1.80, P < 0.05) in NPCs treated with TNF-α, and as a result, 4 potential targeted genes (DDIT4, SBK1, BCL2L11, NRG1) were identified (Fig. 6a). We paid special attention to DDIT4, which has been previously reported to be involved in several ageing-related diseases [[38], [39], [40], [41], [42]]. The Western blot results revealed that DDIT4 expression was significantly higher in degenerative NP tissues (Fig. 6b) and senescent NPCs treated with TNF-α (Fig. 6c) than in normal NP tissues and control NPCs, respectively. Moreover, RIP analysis experiments were conducted using an antibody against AGO2 (Fig. 6d), and qRT‒PCR assays revealed that the expression levels of miR-221-3p and DDIT4 were enriched in AGO2-conjugated beads relative to those in the IgG control group; these data indicate that miR-221-3p combined with the AGO2 protein to form the RISC to regulate DDIT4 mRNA levels (Fig. 6e). The putative binding sites between miR-221-3p and DDIT4 were predicted via the TargetScan database (https://www.targetscan.org/vert_80/) (Fig. 6a). To determine whether miR-221-3p directly suppresses DDIT4 expression by targeting the 3′-UTR of its mRNA, a luciferase reporter plasmid containing a mutation in the 3′-UTR of DDIT4 was constructed, and a plasmid containing the wild-type 3′-UTR of DDIT4 was used as a negative control. The results of luciferase reporter assay showed that miR-221-3p overexpression significantly suppressed the luciferase activity of WT reporter but not the MUT reporter, suggesting that DDIT4 was directly bound by miR-221-3p via complementary target sites (Fig. 6f). Taken together, our results suggested that DDIT4 was a downstream target of miR-221-3p and that miR-221-3p could regulate DDIT4 expression by binding to the 3′-UTR of DDIT4 mRNA.

### 3.6 Hi-Exos reduced the senescence of NPCs by decreasing DDIT4 expression

To validate the biological roles of DDIT4 in the senescence of NPCs, siRNAs targeting DDIT4 were transfected into NPCs, and Western blots were used to confirm the transfection efficacy, and the protein level of DDIT4 was significantly reduced after the transfection of siRNAs targeting DDIT4 (Fig. 6g). We conducted Western blots, EdU staining, and SA-β-gal staining assays to explore the effects of DDIT4 on senescent NPCs. Our results revealed that DDIT4 knockdown significantly reduced p21 protein level (Fig. 6h), promoted cell proliferation (Fig. 6i and j), and decreased SA-β-gal activity (Fig. 6k and l) in senescent NPCs. Therefore, DDIT4 knockdown could alleviate the senescence of NPCs induced by TNF-α. The Western blots showed that protein level of DDIT4 was significantly increased after the transfection of overexpression vector of DDIT4 in NPCs (Fig. 6m). To further verify that Hi-Exos reduce the senescence of NPCs by reducing DDIT4 expression, rescue experiments involving Hi-Exos and DDIT4 were conducted. The results showed that Hi-Exos could significantly decrease p21 protein levels (Fig. 6n), facilitate cell proliferation (Fig. 6o and p), and reduce SA-β-gal activity (Fig. 6q and r) in senescent NPCs; however, the effects of Hi-Exos were obviously attenuated when the DDIT4 overexpression vector was cotransfected. Our findings indicated that Hi-Exos could distinctly reduce the senescence of NPCs, while the overexpression of DDIT4 impaired these protective effects. Taken together, our findings showed that Hi-Exos could reduce the senescence of NPCs by transferring miR-221-3p to decrease DDIT4 expression in senescent NPCs.

### 3.7 Hi-Exos reduced the senescence of NPCs through the miR-221-3p/DDIT4/NF-κB signalling pathway

**Fig. 7:**
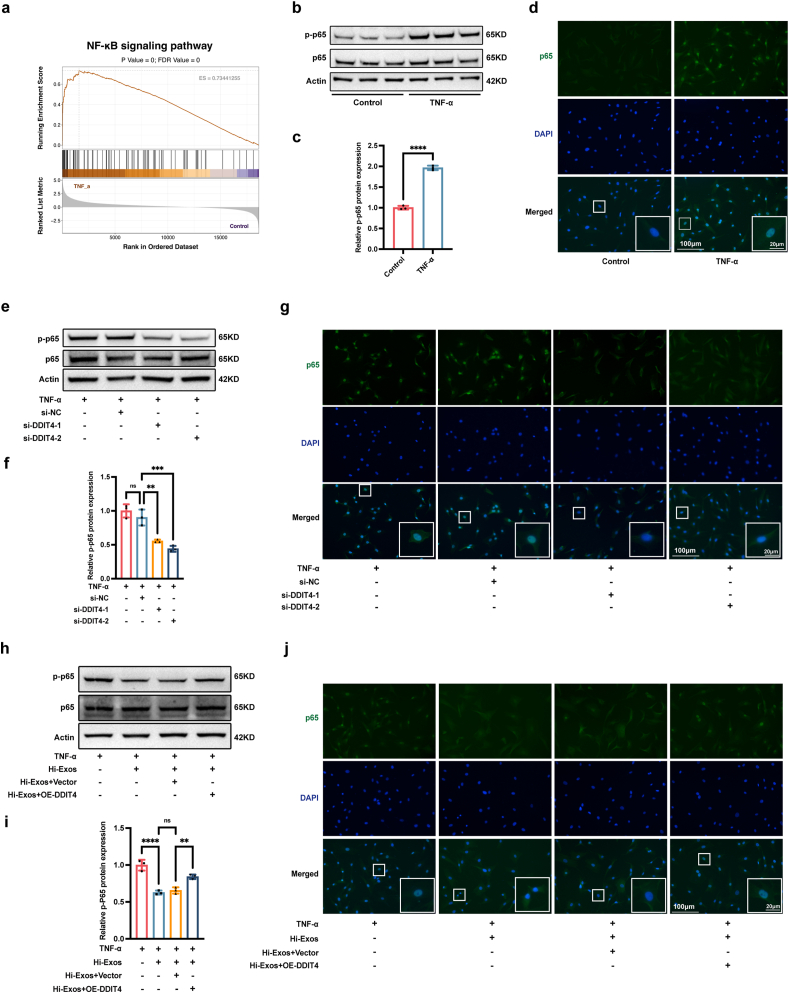
Hi-Exos reduced the senescence of NPCs through the miR-221-3p/DDIT4/NF-κB signalling pathway. a, GSEA enrichment of NF-κB signalling pathway; b-c, Western blots to detect the protein level of p-p65 and p65; d, Immunofluorescence to detect the nuclear translocation of p65; e-f, Western blots to detect the protein level of p-p65 and p65 after the knockdown of DDIT4; g, Immunofluorescence to detect the nuclear translocation of p65 after the knockdown of DDIT4; h-i, Western blots to detect the protein level of p-p65 and p65 in rescue experiments between Hi-Exos and DDIT4 overexpression; j, Immunofluorescence to detect the nuclear translocation of p65 in rescue experiments between Hi-Exos and DDIT4 overexpression. ns, not significant (P < 0.05); ∗∗P < 0.01, ∗∗∗P < 0.001, and ∗∗∗∗P < 0.0001.

The NF-κB signalling pathway has been widely shown to induce the senescence of NPCs by forming an inflammatory microenvironment [43]. GSEA revealed that the NF-κB signalling pathway was activated in senescent NPCs treated with TNF-α (Fig. 7a). Western blot assays revealed that the p-p65/p65 ratio was significantly greater in senescent NPCs treated with TNF-α than that in normal NPCs (Fig. 7b and c). In addition, immunofluorescence revealed that p65 translocated from the cytoplasm into the nucleus in senescent NPCs treated with TNF-α (Fig. 7d). Therefore, the NF-κB signalling pathway was activated in senescent NPCs. DDIT4 was reported to contribute to several human diseases via the activation of the NF-κB signalling pathway [44]. We speculated that the knockdown of DDIT4 could inhibit the NF-κB signalling pathway to improve the inflammatory environment. To verify our speculation, we transfected senescent NPCs with siRNAs targeting DDIT4 to reduce the DDIT4 expression and then assessed the status of the NF-κB signalling pathway. Western blot assays revealed that the p-p65/p65 ratio was significantly reduced in senescent NPCs after the knockdown of DDIT4 (Fig. 7e and f), and immunofluorescence experiments revealed that the nuclear translocation of p65 in senescent NPCs was relieved by DDIT4 knockdown (Fig. 7g). Taken together, our findings showed that the knockdown of DDIT4 obviously inhibited the activation of the NF-κB signalling pathway in senescent NPCs. To verify the role of the Hi-Exos/miR-221-3p/DDIT4/NF-κB signalling pathway in IDD, senescent NPCs were treated with Hi-Exos or DDIT4 overexpression vector. Western blot assays revealed that Hi-Exos reduced the p-p65/p65 ratio in senescent NPCs, whereas this effect was attenuated by the overexpression of DDIT4 (Fig. 7h). Similarly, immunofluorescence experiments revealed that Hi-Exos significantly delayed the nuclear translocation of p65 in senescent NPCs, but the overexpression of DDIT4 reversed the protective effects of Hi-Exos (Fig. 7i and j). Taken together, our results showed that Hi-Exos inhibited the activation of the NF-κB signalling pathway by transferring the miR-221-3p to reduce the DDIT4 expression.

### 3.8 Hi-Exos ameliorated IDD development in the rat IDD model

**Fig. 8:**
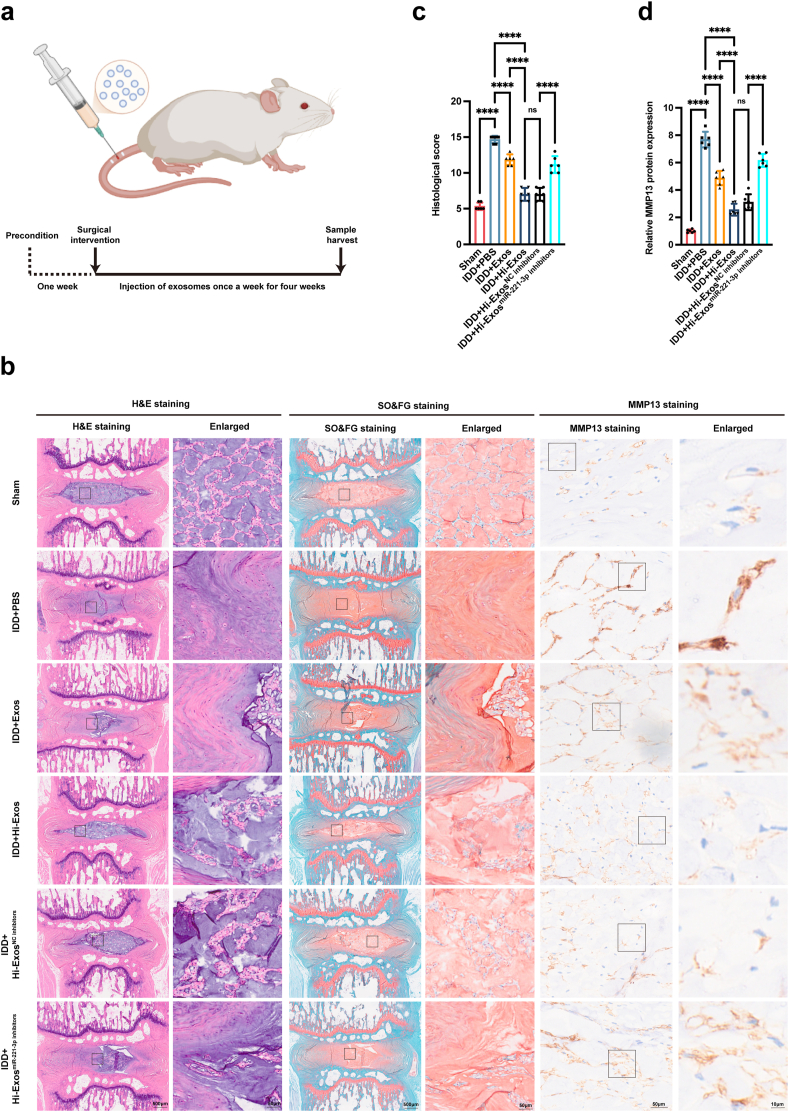
Hi-Exos alleviated the *in vivo* rat IDD through transferring the miR-221-3p to senescent NPCs. a, Schematic diagram of *in vivo* rat IDD experiments; b, H&E, SO&FG, and immumohistochemical staining (MMP13) of rat NP tissues after different exosome interventions for rat IDD; c,d, Histological score (c) and MMP13 protein level (d) of rat NP tissues after different exosome interventions for rat IDD. ∗∗∗∗P < 0.0001.

To further elucidate the protective efficacy of Hi-Exos in mitigating IDD, a rat IDD model was established (Fig. 8a). Histomorphological alterations in nucleus pulposus tissues were evaluated using H&E staining as well as SO&FG staining (Fig. 8b). In the control group, the nucleus pulposus exhibited an oval shape with an intact disc structure and abundant glycosaminoglycan content in the midsagittal cross-section. In contrast, the IDD group displayed a marked reduction in nucleus pulposus area and significant fibrous tissue infiltration, confirming the successful establishment of the *in vivo* IDD model. Administration of either Exos or Hi-Exos significantly attenuated the degenerative changes in intervertebral disc tissues. Notably, the histological score of the Hi-Exos group was significantly lower than that of the Exos group, suggesting that Hi-Exos possess a superior reparative capacity compared to Exos in the context of IDD (Fig. 8c). Similarly, the expression of the senescence-associated marker MMP13 was significantly elevated in the IDD group compared to the Sham group. Both Hi-Exos and Exos treatments significantly reduced the protein levels of MMP13; however, the reduction was more pronounced following Hi-Exos treatment compared to Exos treatment (Fig. 8d). To further investigate the pivotal role of miR-221-3p in Hi-Exos, we administered Hi-Exos^NC inhibitors^ or Hi-Exos^miR−221−3p inhibitors^ to treat rat IDD. Histological analyses via H&E (Fig. 8b), SO&FG (Fig. 8c), and MMP13 (Fig. 8d) staining demonstrated that Hi-Exos^NC inhibitors^ effectively repaired IDD-induced damage, however, the reparative capacity of Hi-Exos was significantly diminished following the knockdown of miR-221-3p. Therefore, our findings indicated that Hi-Exos were more effective than Exos in alleviating IDD, and that the reparative effects of Hi-Exos were mediated, at least in part, through the transfer of miR-221-3p.

**Fig. 9:**
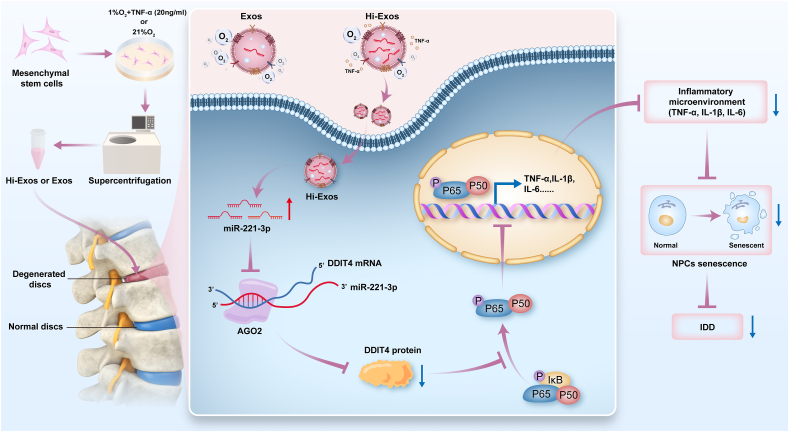
Schematic diagram of Hi-Exos in relieving the senescence of NPCs and IDD. Hi-Exos was superior to Exos in relieving the senescence of NPCs and IDD for the increased epigenetic factor miR-221-3p level. In details, Hi-Exos transferred the miR-221-3p to the senescent NPCs to decrease the DDIT4 protein level in senescent NPCs, which further inhibited the activation of NF-κB signalling pathway to relieve the senescence of NPCs and repair the IDD.

In summary, the hypoxic and inflammatory environments increased the miR-221-3p level in Hi-Exos. Hi-Exos reduced the senescence of NPCs via epigenetic regulation mediated by miR-221-3p that was transferred into senescent NPCs, after which exosomal miR-221-3p reduced DDIT4 expression to inhibit the NF-κB signalling pathway (Fig. 9). Therefore, Hi-Exos constituted a potential therapeutic strategy for IDD treatment.

## 4 Discussion

Senescence of NPCs induced by inflammatory factors (e.g., TNF-α) is an important pathogenic factor of IDD, which leads to low back pain and incontinence [1,6]. Although previous studies have shown that MSC-derived exosomes have good repair activity in the treatment of IDD [13], no study has explored whether exosomes derived from MSCs exposed to hypoxic and inflammatory environments have a better ability to relieve the senescence of NPCs than normal exosomes do. In this study, we found that, compared with control Exos, H-Exos, and I-Exos, Hi-Exos had more ability to repair damage from IDD. Further exploration revealed that Hi-Exos alleviated NPC senescence by introducing miR-221-3p to the cells; through epigenetic mechanisms, this miRNA decreased DDIT4 expression and inhibited the activation of the NF-κB signalling pathway.

MSC-derived exosomes show promise for application in the treatment of several ageing-related diseases, including IDD [13]. MSC-derived exosomes can repair IDD by reducing oxidative stress, attenuating mitochondrial dysfunction [45], inhibiting apoptosis [46] or pyroptosis [47], promoting autophagy [48], and alleviating cell senescence [49]. To further improve the repair capacity of MSC-derived exosomes in IDD, researchers have tried to use different strategies, such as static magnetic fields [50], quercetin [51], and hypoxia [14], to pretreat MSCs. Hypoxia is the most common pretreatment strategy for MSC-derived exosomes. Many studies have shown that hypoxia pretreatment of MSCs can improve the biological functions of exosomes by changing the cargos in the exosomes [15,17,19,28], possibly because most MSCs are in a hypoxic ecological niche *in vivo,* and culturing MSCs in a low-oxygen environment is more closely aligned with their physiological environment [19]. Another popular pretreatment strategy for MSCs involves the use of inflammatory factors, such as TNF-α and lipopolysaccharide [16,18]. Previous studies have shown that pretreatment of MSCs with inflammatory factors could improve the repair functions of MSCs by increasing their anti-inflammatory capacity [16,18]. Considering that the inflammatory microenvironment is the main factor involved in the senescence of NPCs in IDD, we speculated that the addition of the inflammatory cytokine TNF-α to MSCs exposed to hypoxia might further improve the repair ability of exosomes, resulting in the reduction of senescence of NPCs. Our findings, for the first time, revealed that Hi-Exos derived from MSCs exposed to hypoxic and inflammatory environments exhibited better repair functions than normal Exos did in attenuating the senescence of NPCs, and the *in vivo* experiments also indicated that Hi-Exos could better repair damage from IDD in an *in vivo* rat IDD model.

Many studies have reported that MSC-derived exomes repaired IDD through the transfer of miRNAs to NPCs [13,46,52,53]. Su et al. reported that MSC-derived exosomes alleviated IDD through the transfer of miR-145a-5p to these cells to regulate the expression of USP31 [52]. Duan et al. reported that MSC-derived exosomes inhibited NPC apoptosis via the transfer of miR-125b-5p to the cells to regulate the TRAF6/NF-κB signalling pathway [46]. Chen et al. reported that MSC-derived exosomes attenuated NPC apoptosis by transferring miR-155-5p to the cells to decrease TRIM32 expression [54]. Yuan et al. reported that MSC-derived exosomes inhibited NPC pyroptosis by transferring miR-26a-5p to the cells to inhibit the METTL14/NLRP3 signalling pathway [53]. In the present study, we detected a significant difference in the miRNA expression profile between the Hi-Exos and Exos, which indicated that the better repair functions of the Hi-Exos might be due to alterations in the miRNAs found in the exosomes. We paid special attention to miR-221-3p because it was significantly decreased in senescent NPCs compared with normal NPCs but increased in Hi-Exos compared with Exos. Previous studies have reported the protective roles for miR-221-3p in IDD [55,56]. Zhang et al. reported that miR-221-3p could ameliorate the pyroptosis of NPCs by reducing PTEN expression [55]. Ching-Hua Yeh et al. reported that miR-221-3p was significantly decreased in annulus fibrosus cells in IDD and that the overexpression of miR-221-3p attenuated the osteogenic differentiation of annulus fibrosus cells to inhibit IDD [56]. In our study, we discovered that miR-221-3p was significantly decreased in senescent NPCs treated with TNF-α, which indicated that the absence of miR-221-3p contributed to the senescence of NPCs. Furthermore, knockdown of miR-221-3p in Hi-Exos reduced the protective effects of Hi-Exos in relieving the senescence of NPCs and *in* an *in vivo* rat IDD model. Therefore, our findings revealed that the epigenetic regulator miR-221-3p is a promising therapeutic target for IDD and that Hi-Exos repair damage form IDD through the transfer of miR-221-3p to senescent NPCs.

Epigenic modifications play important roles in both maintaining human health and promoting disease progression. miRNAs, as epigenetic regulators, can regulate gene expression by binding to complementary sequences of the 3′-UTRs of targeted genes [57]. In the present study, bioinformatics analysis and sequencing data revealed that DDIT4 is the downstream effector of exosomal miR-221-3p. DDIT4, also known as REDD1, is an evolutionarily conserved protein that is usually overexpressed when cells are subjected to various stress conditions, such as oxidative stress and hypoxia [58]. DDIT4 is reportedly associated with various ageing-related diseases, such as Alzheimer's disease [59], osteoarthritis [60], and IDD [42,61]. DDIT4 has been reported to contribute to IDD by promoting NPC pyroptosis [42] or apoptosis [61]. In our study, we observed a significant increase in DDIT4 in senescent NPCs induced by TNF-α, and the knockdown of DDIT4 via siRNA treatment alleviated the senescence of NPCs. The NF-κB signalling pathway has been widely demonstrated to play important roles in IDD by promoting the expression of inflammatory cytokines (e.g., TNF-α, IL-6, and IL-1β), which induce inflammatory conditions [43]. Previous studies have shown that DDIT4 promotes inflammation by activating the NF-κB signalling pathway in several diseases; however, whether DDIT4 contributes to the senescence of NPCs through the activation of the NF-κB signalling pathway remains unclear. In our study, we observed that the NF-κB signalling pathway was activated in TNF-α-stimulated senescent NPCs; however, activation of the NF-κB signalling pathway could be inhibited through DDIT4 knockdown. Furthermore, rescue experiments demonstrated that Hi-Exos prevented the activation of the NF-κB signalling pathway by reducing DDIT4 expression in senescent NPCs.

## 5 Conclusions

In conclusion, we discovered that pretreatment of MSCs by exposure to hypoxic and inflammatory environments significantly enhanced the repair capacity of exosomes. With respect to the molecular mechanism, Hi-Exos were able to reduce the senescence of NPCs via the introduction of miR-221-3p to inhibit the DDIT4/NF-κB signalling pathway. Taken together, our *in vitro* and *in vivo* findings indicated that Hi-Exos could be an effective intervention for attenuating the senescence of NPCs in patients with IDD.

## CRediT authorship contribution statement

**Yongzhao Zhao:** Writing – review & editing, Writing – original draft, Software, Methodology, Investigation, Formal analysis, Data curation, Conceptualization. **Longting Chen:** Writing – original draft, Software, Methodology, Investigation, Formal analysis, Data curation. **Shuai Jiang:** Writing – original draft, Validation, Investigation, Data curation. **Zhenquan Wu:** Writing – review & editing, Software, Methodology, Formal analysis, Data curation. **Qian Xiang:** Writing – review & editing, Visualization, Investigation. **Jialiang Lin:** Writing – original draft, Software, Methodology. **Shuo Tian:** Writing – review & editing, Validation, Resources, Investigation, Data curation. **Zhuoran Sun:** Writing – review & editing, Visualization, Supervision, Formal analysis, Data curation. **Chuiguo Sun:** Writing – review & editing, Supervision, Investigation. **Weishi Li:** Writing – review & editing, Supervision, Investigation, Funding acquisition.

## Ethics approval and consent to participate

All animal experimentation was endorsed by the Institutional Animal Care and Use Committee at Peking University Health Science Center. Ethical approval was obtained from the Ethics Committee of Peking University Third Hospital (approval number LA2022421), and informed consent was obtained from all patients involved in this study.

## Data availability

The data generated during the current study are available from the corresponding author upon reasonable request.

## Declaration of competing interest

The authors declare no competing interests.
