# Supplementary material for: Exosomes derived from MSCs exposed to hypoxic and inflammatory environments slow intervertebral disc degeneration by alleviating the senescence of nucleus pulposus cells through epigenetic modifications
Source: Bioact Mater. 2025 Mar 20;49:515–30. doi: 10.1016/j.bioactmat.2025.02.046 (PMC11979484; doi:10.1016/j.bioactmat.2025.02.046)
Supplement: Multimedia component 1 [file mmc1.docx]

**Supplementary Table 1. Details of siRNAs, miRNA mimics or inhibitors, primers used in this study.**

| **siRNAs** | **Sequences** |
| --- | --- |
| **Rat si-DDIT4-1** |  |
| *Forward* | 5’-AGGCAAGAGCUGCCAUAGU-3’ |
| *Reverse* | 5’-ACUAUGGCAGCUCUUGCCU-3’ |
| **Rat si-DDIT4-2** |  |
| *Forward* | 5’-CUGUUGAGUUCUGCCAACU-3’ |
| *Reverse* | 5’-AGUUGGCAGAACUCAACAG-3’ |
| **Rat si-AGO2-1** |  |
| *Forward* | 5’-CGAAGUGCAAGUUUCAAUATT-3’ |
| *Reverse* | 5’-UAUUGAAACUUGCACUUCGTT-3’ |
| **Rat si-AGO2-2** |  |
| *Forward* | 5’-GGAGAACAAUCAAACUACATT-3’ |
| *Reverse* | 5’-UGUAGUUUGAUUGUUCUCCTT-3’ |
| **Rat si-NC** |  |
| *Forward* | 5’-UUCUCCGAACGUGUCACGUTT-3’ |
| *Reverse* | 5’-ACGUGACACGUUCGGAGAATT-3’ |
|  |  |
| **miRNA mimics**  **or inhibitors** |  |
| miR-221-3p mimics | 5’-AGCUACAUUGUCUGCUGGGUUUC-3’ |
| miR-221-3p inhibitors | 5’-GAAACCCAGCAGACAATGTAGC-3’ |
|  |  |
| **Primers** |  |
| **miR-221-3p** | 5’-GAGCTACATTGTCTGCTGGGTTTC-3’ |
| **Rat U6** |  |
| *Forward* | 5’-CTCGCTTCGGCAGCACATA-3’ |
| *Reverse* | 5’-CGAATTTGCGTGTCATCCT-3’ |
| **Human U6** |  |
| *Forward* | 5’-CTCGCTTCGGCAGCACA-3’ |
| *Reverse* | 5’-AACGCTTCACGAATTTGCGT-3’ |
| **Rat DDIT4** |  |
| *Forward* | 5’-CTGGTGCTGCGTCTGGACTC-3’ |
| *Reverse* | 5’-GCGTCAGGGACTGGCTGTAAC-3’ |
| **Rat GAPDH** |  |
| *Forward* | 5’-AACAGCAACTCCCATTCTTCC-3’ |
| *Reverse* | 5’-TGGTCCAGGGTTTCTTACTCC-3’ |

NC, negative control.
